# Engineering of *Saccharomyces cerevisiae* for the production of poly-3-d-hydroxybutyrate from xylose

**DOI:** 10.1186/s13568-015-0100-0

**Published:** 2015-02-25

**Authors:** Anders G Sandström, Alejandro Muñoz de las Heras, Diogo Portugal-Nunes, Marie F Gorwa-Grauslund

**Affiliations:** Division of Applied Microbiology, Department of Chemistry, Lund University, PO Box 124, Lund, SE-221 00 Sweden

**Keywords:** Polyhydroxyalkanoate, PHB, Xylose, *Saccharomyces cerevisiae*, Metabolic engineering, Biopolymer

## Abstract

Poly-3-d-hydroxybutyrate (PHB) is a promising biopolymer naturally produced by several bacterial species. In the present study, the robust baker’s yeast *Saccharomyces cerevisiae* was engineered to produce PHB from xylose, the main pentose found in lignocellulosic biomass. The PHB pathway genes from the well-characterized PHB producer *Cupriavidus necator* were introduced in recombinant *S. cerevisiae* strains already capable of pentose utilization by introduction of the fungal genes for xylose utilization from the yeast *Scheffersomyces stipitis*. PHB production from xylose was successfully demonstrated in shake-flasks experiments, with PHB yield of 1.17 ± 0.18 mg PHB g^−1^ xylose. Under well-controlled fully aerobic conditions, a titer of 101.7 mg PHB L^−1^ was reached within 48 hours, with a PHB yield of 1.99 ± 0.15 mg PHB g^−1^ xylose, thereby demonstrating the potential of this host for PHB production from lignocellulose.

## Introduction

Petroleum-based plastics are essential elements of our contemporary society, and will remain so for a foreseeable future. Still, the world is standing at the threshold of an era marked by scarce fossil-based resources (Lambert et al. 2014; Foley et al. 2011), which called for a transition towards the production of bio-based bulk chemicals from renewable feedstock (European Parliament CotEU 2009). Among candidate feedstock for bio-based polymer production, lignocellulosic biomass such as corn stover, wheat straw or sugar cane bagasse, are amongst the most promising ones (Gírio et al. 2010). Another readily available lignocellulosic feedstock is the spent sulfite liquor (SSL), one of the main by-products from the pulp and paper industry (Novy et al. 2013).

However, up to now, the bio-based polymers that are entering the market as replacement for their petroleum-based counterparts are produced from simple sugars, such as glucose, or food crop residuals such as glycerol. Polyhydroxyalkanoates (PHAs) are some of the most promising biodegradable biopolymers for a sustainable future (Philip et al. 2007). Poly-(*R*)-3-hydroxybutyrate (PHB), that is naturally produced by several bacterial species as an energy storage depot, typically under conditions of nitrogen starvation (Suriyamongkol et al. 2007; Madison and Huisman 1999), is one of the most studied PHAs. Numerous hosts have been suggested as PHA producers, such as natural PHB-producing bacterial species or engineered *Escherichia coli.* Nevertheless *E. coli* suffers from regular phage infections in industrial production settings (Lian et al. 2014), while the natural PHB-producing bacterial species are not well adapted to harsh environments, such as growth in SSL. Instead baker’s yeast *Saccharomyces cerevisiae,* that is known for its tolerance to acidic and inhibitor-rich conditions, is considered one of the favored species for converting the sugars found in SSL into bulk chemicals of interest – e.g. PHB (Demeke et al. 2013). SSLs are commonly rich in xylose, a pentose sugar that is not naturally consumed by *S. cerevisiae*. Therefore, efficient xylose-metabolizing *S. cerevisiae* strains have been developed (Hahn-Hägerdal et al. 2007; Kim et al. 2013).

The potential of engineered *S. cerevisiae* as a producer of PHAs from pure hexose sugars has previously been explored by several groups. First, only the polyhydroxyalkanoate synthase gene from the PHB producer *Cupriavidus necator* (formerly known as *Ralstonia eutropha* (Vandamme and Coenye 2004)) was overexpressed (Leaf et al. 1996) and PHB was produced by the available cytosolic 3-hydroxybutyryl-CoA, derived from the fatty acid β–oxidation pathway. Several strategies aiming at increasing the available pool of precursors have been tested in *S. cerevisiae* in order to increase the yield of PHB, for example addition of the *C. necator* β-ketothiolase and acetoacetyl-CoA reductase genes (Carlson and Srienc 2006; Breuer et al. 2002), enzymes for boosting ethanol assimilation and acetyl-CoA synthesis (Kocharin et al. 2012) and addition of the phosphoketolase pathway and non-phosphorylating NADP^+^-dependent glyceraldehyde-3-phosphate dehydrogenase for increasing the NADPH pool (Kocharin et al. 2013). Here, we demonstrate that *S. cerevisiae* can also be engineered to produce PHB from d-xylose.

## Materials and methods

### Strains, media and culture conditions

Plasmids and yeast strains used in this study are listed in Table 1. *E. coli* strain NEB5-α (New England Biolabs) was used for sub-cloning of plasmid DNA and further propagation. Lysogeny broth (LB) (5 g L^−1^ yeast extract, 10 g L^−1^ peptone, 5 g L^−1^ NaCl, pH 7.0) medium was used for routine culturing of *E. coli* and 50 mg L^−1^ ampicillin was added to LB when needed. Bacterial transformants were selected on solid LB plates (15 g L^−1^ agar), supplemented with ampicillin (50 mg L^−1^), for 16 h at 37°C. Cultures of vector-carrying *E. coli* were recovered from 25% glycerol stocks stored at −80°C and grown in liquid LB medium, supplemented with ampicillin, for 14–16 h at 37°C and 180 rpm in an orbital shaker.

**Table 1:** **Plasmids and strains used in this study**

**Plasmids**	**Relevant genotype**	**Reference**
YIplac128	*LEU2*	(Gietz and Akio 1988)
YIpOB8	*pTDH3*-*XYL1-tADH1*; *pPGK1-XYL2-tPGK1*; *URA3*	(Runquist et al. 2010)
YIpAGS2	YIplac128; *pTEF1*-*PhaA*-*tTEF1*; *pGPM1*-*PhaB1*-*pGPM1*; *pTPI1*-*PhaC1*-*tTPI1*; *LEU2*	This study
**Yeast strains**	**Relevant genotype**	**Reference**
TMB 3043	CEN.PK2-1C; *gre3*Δ; *his3*::*pPGK1-XKS1-tPGK1*, *HIS3*; *tal1*::*pPGK1-TAL1*-*tPGK1*; *tkl1*::*pPGK1-TKL1-tPGK1*; *rki1*::*pPGK1-RKI1-tPGK1*; *rpe1*::*pPGK1-RPE1-tPGK1*; *ura3*, *leu2*	(Karhumaa et al. 2005)
TMB 4440	TMB 3043; *ura3*::YIpOB8; *leu2*	This study
TMB 4443	TMB 4440; *leu2*::YIpAGS2	This study
TMB 4444	TMB 4440; *leu2*::YIplac128	This study

Yeast strains were recovered from 20% glycerol stocks stored at −80°C, by streaking on solid YPD plates (10 g L^−1^ yeast extract, 20 g L^−1^ peptone, 20 g L^−1^ glucose, 15 g L^−1^ agar) for two days at 30°C. Yeast cultures were grown in liquid YPD when preparing the strain for transformation for 14–16 h, or less when required, at 30°C and 180 rpm in an orbital shaker.

Engineered yeast strains were selected on solid YNB medium (6.7 g L^−1^ Yeast Nitrogen Base without amino acids (Becton, Dickinson and Company, USA) supplemented with 20 g L^−1^ glucose or xylose and 15 g L^−1^ agar). Leucine was added for complementation at a concentration of 30 mg L^−1^ when required.

All chemicals were purchased from Sigma-Aldrich if not otherwise stated.

### PHB pathway expression vector construction

The PHB pathway genes *PhaA*, *PhaB1*, and *PhaC1* from *C. necator* (Poehlein et al. 2011) were codon optimized towards *S. cerevisiae* and synthesized (Eurofins Genomics, Germany). The codon-optimized genes have been deposited with following accession numbers; *PhaA* [GenBank:KP681582], *PhaB1* [GenBank:KP681583], *PhaC1* [GenBank:KP681584]. The three coding regions were inserted in-between the strong constitutive promoter-terminator pairs of the genes *TEF1*, *TPI1*, *GPM1* (Sun et al. 2012), respectively. The promoters and terminators were amplified directly from the genome of *S. cerevisiae* CEN.PK113-7D (Nijkamp et al. 2012), and the PHB pathway genes were amplified by PCR from respective vector. The fragments were amplified with Phusion Hotstart II polymerase (Thermo Scientific, USA) using the following PCR program: initial denaturation at 98°C for 30 s, 30 cycles of denaturation at 98°C for 10 s, annealing at 65°C (−0.5°C∕cycle) for 30 s and elongation at 72°C for 15 s or 1 min, and a final elongation step for 10 min at 72°C. The three purified promoter, coding sequence and terminator fragments were assembled by overlap extension PCR (OE-PCR). Equimolar fragment amount were mixed, and the following overlap-extension program was used; 98°C for 30 s, then 16 cycles at 98°C for 10 s, 68°C (−0.5°C/cycle) for 30 s, 72°C for 1 min and then hold at 12°C for the addition of amplification primers (50 μM each), whereafter the program continued at 98°C for 30 s, then 20 cycles at 98°C for 30 s, 68°C (−0.5°C/cycle) for 30 s, 72°C for 1 min, and then a final elongation at 72°C for 10 min. The primers were designed such that the flanking ends of the generated fragments overlapped with the neighboring regions, enabling the use of In-Fusion cloning (Clontech, Takara-Bio, Japan). Linear vector fragments of YIplac128 (Gietz and Akio 1988) were generated by double-digestion with Fast Digest *Cfr*9I (*Xma*I) and *Ssp*DI (*Kas*I) (Thermo Scientific, USA). The digested vector, and the three promoter-gene-teminator fragments were mixed in equimolar amounts and were assembled by In-Fusion cloning, according to the supplier’s instruction. High efficiency competent NEB5-α cells were transformed according to the supplier’s instructions. Plasmids were propagated, a diagnostic restriction digestion was performed and the cloned region was fully sequenced. The oligonucleotides used for the PCRs are listed in Table 2.

**Table 2:** **Primers used in the present study**

**Name**	**Amplification target**	**Sequence (5‘ to 3’)**
TEF1p_f	*S. cerevisiae TEF1* promoter	CTAGAGGATCCCCGGGTATAGCTTCAAAATGTTTCTAC
TEF1p_r		CACGTCAGTCATTTTGTAATTAAAACTTAGATTAG
PhaA_f	Codon optimized *PhaA* ORF from *C. necator*	GTTTTAATTACAAAATGACTGACGTGGTGATTG
PhaA_r		CACGTCAGTCATTTTGTAATTAAAACTTAGATTAGATTG
TEF1t_f	*S. cerevisiae TEF1* terminator	GTTGAAAGAAAGTAAGGAGATTGATAAGACTTTTC
TEF1t_r		GATATAGAGCTCAGATAGCGCCGATCAAAG
TPI1p_f	*S. cerevisiae TPI1* promoter	TCTGAGCTCTATATCTAGGAACCCATCAG
TPI1p_r		CAATCCTTTGCGTCATTTTTAGTTTATGTATGTGTTTTTTG
PhaB1_f	Codon optimized *PhaB1* ORF from *C. necator*	CATACATAAACTAAAAATGACGCAAAGGATTGCGTATG
PhaB1_r		GATAATATTTTTATATAATTATATTATTAACCCATGTGTAAGCCAC
TPI1t_f	*S. cerevisiae TPI1* terminator	CACATGGGTTAATAATATAATTATATAAAAATATTATCTTCTTTTC
TPI1t_r		CTAACTAGTTATATAACAGTTGAAATTTGGATAAG
GPM1p_f	*S. cerevisiae GPM1* promoter	TATATAACTAGTTAGTCGTGCAATGTATGAC
GPM1p_r		CTTTACCAGTGGCCATTATTGTAATATGTGTGTTTGTTTG
PhaC1_f	Codon optimized *PhaC1* ORF from *C. necator*	CATATTACAATAATGGCCACTGGTAAAGG
PhaC1_r		CATTCATTCTTCAGACTTATGCCTTTGCTTTCACATAC
GPM1t_f	*S. cerevisiae GPM1* terminator	GAAAGCAAAGGCATAAGTCTGAAGAATGAATGATTTG
GPM1t_r		TACCGCATCAGGCGCCATATTCGAACTGCCCATTCAG

### Yeast strain engineering

*S. cerevisiae* TMB 3043 (Karhumaa et al. 2005) was transformed with the *Eco*RV-linearized vector YIpOB8 (Runquist et al. 2010), generating the strain TMB 4440. The yeast cells were transformed using the high-efficiency Li-Ac method (Gietz and Schiestl 2007), and plated on YNB-xylose (20 g L^−1^)-plates, supplemented with leucine (30 mg L^−1^). The strain was subsequently transformed with *Eco*9I-linearized YIpAGS2 or YIp128 – to form the PHB-producing strain TMB 4443 or the corresponding control strain TMB 4444 (Table 1). Transformed strains were validated by amplifying stretches of the integrated cassette by PCR, using extracted genomic DNA as template and primers found in Table 2.

### Aerobic cultivations

For the shake flasks experiments, well-aerated baffled shake flasks were used. Pre-cultures were started by transferring an isolated colony into 5.0 mL of defined buffered YNB-xylose (13.4 g L^−1^ Yeast nitrogen base, 50 g L^−1^ xylose, 50 mM potassium hydrogen phthalate, pH 5.5) in 50 mL conical centrifuge tube, shaking at 30°C at 180 rpm overnight. 50 mL buffered YNB-xylose was inoculated with the pre-culture to a starting OD_620_ of 0.05, in a 250 mL baffled shake flask. The cultures were grown at 30°C at 180 rpm for at least 82 hours. Optical density during growth was monitored at 620 nm using an Ultrospec 2100 Pro spectrophotometer (Amersham Biosciences Corp., USA). Samples were extracted for PHB quantification, OD_620_, cell dry weight measurements, and metabolite analysis, and were done in technical duplicates. Maximum specific growth rate was calculated by the analysis of the exponential growth phase, using a linear regression of the natural logarithm derived from the OD_620_.

For the experiments in bioreactors, unbuffered YNB-xylose (13.4 g L^−1^ Yeast nitrogen base, 50 g L^−1^ xylose) was used as media. Well-controlled Multifors 1.2 L vessels (Infors, Switzerland) were sparged with 1.0 vvm air at 30°C and 300 rpm. A culture volume of 300 mL was inoculated with YNB-xylose freshly grown cells to a starting OD_620_ of 0.05. Silicon antifoam RD emulsion (Dow Corning, USA), 3 M KOH and 3 M H_2_SO_4_ were added as needed in order to avoid the excess of foaming and to keep the pH at 5.5, respectively. Two independent biological replicates were performed for each condition.

### Analysis of biomass and metabolites

Cell dry weight was measured in triplicate by filtering a known volume (3.0 to 5.0 mL) of the culture through a pre-weighed nitrocellulose filter with 0.45 μm pore size (PALL, USA). The filters were washed, dried and weighed after equilibrating to room temperature in a desiccator. Biomass was correlated to OD_620_ by a single point calibration, based on the final measuring time point. Concentrations of glucose, xylose, xylitol, glycerol, acetate and ethanol were analyzed by HPLC (Waters, USA). Metabolites were separated using an Aminex HPX-87H ion exchange column (Bio-Rad, USA) at 45°C with 5 mM H_2_SO_4_ as mobile phase, at a flow rate of 0.6 mL min^−1^. All compounds were detected with a RID-10A refractive index detector (Shimadzu, Japan). Concentrations of metabolites were calculated from an external seven-point standard calibration curve. Samples were analyzed in technical duplicates.

### PHB quantification

The method used relied on the quantitative conversion of PHB to crotonic acid catalyzed by hot concentrated sulfuric acid (Law and Slepecky 1961). A culture volume of 0.5 or 1.0 mL was harvested and transferred to a 1.5 mL microcentrifuge tube. The harvested cells were centrifuged for 5 min at 6000 × *g* and the supernatant was discarded by careful aspiration (FTA-1, Biosan, Latvia). The pellet was washed with 1.0 mL ultrapure H_2_O and resuspended by vortexing, centrifuged for 5 min at 6000 × *g* and the supernatant was discarded. This wash step was repeated two times. H_2_SO_4_ (0.5 mL, 96% v/v) was added to the pellet. The pellet was resuspended by careful pipetting, and the open-lid tubes were transferred to a heat block (Grant QBD1, Grant Instruments, UK), and incubated at 95°C for 1 h. The resulting solution was diluted 20 times by a serial dilution and further analyzed by HPLC, using the same conditions as for the analysis of metabolites, and quantified by comparing to an external calibration curve of crotonic acid. Commercially available PHB (#363502, Sigma-Aldrich) was used as method control and was processed similarly as pelletized cells. Samples were analyzed in technical duplicates.

## Results

### Construction of the expression vector carrying the PHB pathway genes

**Figure 1:**
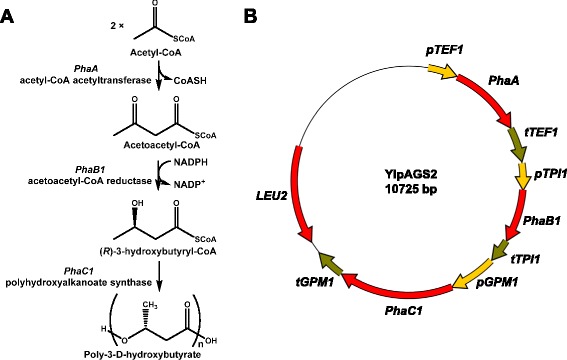
**The PHB metabolic pathway, consisting of A) metabolite structures, involved enzymes, cofactors and B) the genes and regulatory sequences in the integrative vector YIpAGS2 in a schematic representation.**

The *C. necator* PHB pathway, consisting of a β-ketothiolase (acetyl-CoA acetyltransferase) (encoded by *PhaA*), an acetoacetyl-CoA reductase (encoded by *PhaB1*), and a PHB synthase (encoded by *PhaC1*) (Figure 1A), was chosen for PHB production in *S. cerevisiae* as it has been successfully used in the past (see e.g. (Carlson and Srienc 2006; Kocharin et al. 2012; Breuer et al. 2002)). The genes, ordered from MWG Eurofins, were codon optimized for *S. cerevisiae*, which notably led to lower GC-content (47–52% GC) than the native bacterial genes (63–68% GC). All three genes were cloned behind a strong constitutive promoter (*pTEF1*, *pTPI1* and *pGPM1* for *PhaA*, *PhaB1* and *PhaC1*, respectively) into a single large vector, named YIpAGS2 (Figure 1B), based on YIplac128 which has a *LEU2* gene for complementation of auxotrophic strains. The entire pathway was sequenced and validated.

### Strain engineering

*S. cerevisiae* strain TMB 3043 (Karhumaa et al. 2005), that has been engineered for increased flux from xylulose to glycolysis, through the overexpression of xylulokinase and the non-oxidative pentose phosphate pathway genes, was transformed with YIpOB8 carrying the *Scheffersomyces stipitis* xylose genes encoding xylose reductase (XR) and xylitol dehydrogenase (XDH) (Bengtsson et al. 2009), generating strain TMB 4440.

TMB 4440 was further transformed with the constructed linearized YIpAGS2 that carries *C. necator* PHB pathway gene or with a linearized integrative control vector (YIplac128), generating strains TMB 4443 (PHB+) and TMB 4444 (PHB-), respectively.

### Evaluation of PHB production from xylose

**Figure 2:**
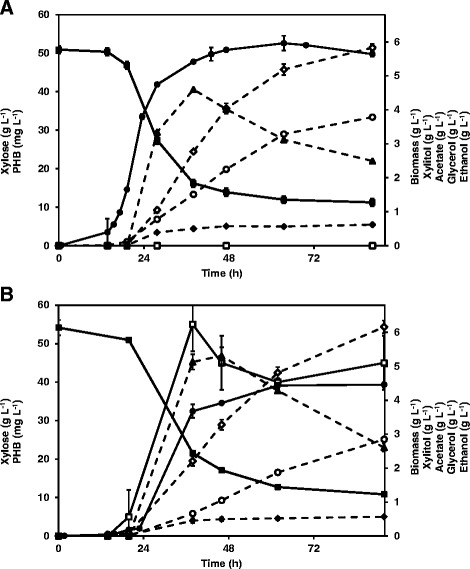
**Representative aerobic growth and metabolite profiles from recombinant** ***S. cerevisiae*** **strains A) TMB 4444 (Control strain, PHB-) and B) TMB 4443 (PHB+), with xylose defined medium in buffered shake flask cultures.** *Legend*: xylose(■); biomass(●); glycerol(♦); ethanol(▲); PHB(□); xylitol(○); acetate(◊).

The generated strains were compared aerobically in well-stirred baffled flasks to evaluate their PHB production capacity. The strains were grown with d-xylose as sole carbon source, until the cultures reached late stationary phase. Metabolites were analyzed and cell dry weight calculated. Representative growth plots for the control strain TMB 4444 and the TMB 4443 strain expressing the PHB pathway genes are displayed in Figure 2A and 2B, respectively.

As expected, both strains were able to grow on xylose. Xylitol, acetate and ethanol were the main by-products (Table 3). However strain TMB 4443 (PHB+) generated less biomass and xylitol and more ethanol than the control strain TMB 4444. This was accompanied by PHB production that appeared to be concomitant with biomass formation. In the early exponential phase, PHB content rose more rapidly than the biomass titers under these shake flask conditions (Figure 2B). PHB yield peaked at 1.17 ± 0.33 mg PHB g^−1^ xylose consumed (Table 3) at the end of the exponential phase and further ethanol consumption did not result in additional PHB accumulation (Figure 2B).

**Table 3:** **Physiological results of the strains grown aerobically on xylose on shake flasks or bioreactors**

**Strain**	**Growth condition**	**Growth rate** ***μ***_**max**_ **(h** ^**−1**^ **)**	**Yields**						**PHB titer (mg/L)**	**Final PHB content (% CDW)**	**Xylose/sugars consumed (%)**
			**Y**_**sx**_ **(g/g Xyl)**	**Y**_**sEtOH**_ **(g/g Xyl)**	**Y**_**sAc**_ **(g/g Xyl)**	**Y**_**sXylitol**_ **(g/g Xyl)**	**Y**_**sGly**_ **(g/g Xyl)**	**Y**_**sPHB**_ **(mg/g Xyl)**			
TMB 4443	Shake flask	0.231 ± 0.01	0.11 ± 0.02	0.10 ± 0.05	0.14 ± 0.0	0.05 ± 0.03	0.01 ± 0.0	1.17 ± 0.18	45.0 ± 3.54	1.04 ± 0.03	76.8 ± 4.6
TMB 4444	Shake flask	0.249 ± 0.01	0.14 ± 0.0	0.06 ± 0.0	0.14 ± 0.0	0.10 ± 0.0	0.02 ± 0.0	0.0 ± 0.0	0.0 ± 0.0	0.0 ± 0.0	80.1 ± 3.0
TMB 4443	Bioreactor	0.184 ± 0.01	0.41 ± 0.02	0.004 ± 0.0	0.0 ± 0.0	0.028 ± 0.0	0.0 ± 0.0	1.99 ± 0.15	101.7 ± 7.1	0.49 ± 0.06	100 ± 0

The values reported are calculated for aerobic cultivations on defined media with xylose (50 g L^−1^) as carbon source. The specific growth rate is calculated for the exponential phase. Yields and titers are calculated from a single time point in the early stationary phase. Reported values represent the mean ± SD of at least two independent cultivations performed. Y_sx_: biomass yield on xylose, Y_sEtOH_: yield of ethanol on xylose, Y_sAc_: yield of acetate on xylose, Y_sXylitol_: yield of xylitol on xylose, Y_sGly_: yield of glycerol on xylose, Y_sPHB_: yield of PHB on xylose. PHB titer: The volumetric PHB titer. PHB/CDW: The PHB component as percentage of total cell dry weight.

**Figure 3:**
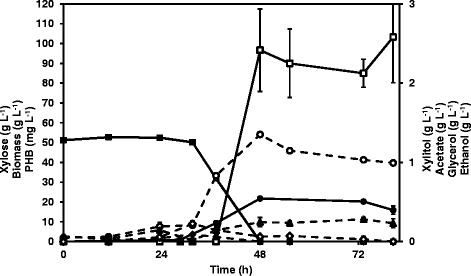
**Representative fully aerobic batch growth and metabolite profile from recombinant** ***S. cerevisiae*** **strain TMB 4443 (PHB+) in xylose defined medium at pH 5.5 using a well-controlled bioreactor.** *Legend*: xylose(■); biomass(●); glycerol(♦); ethanol(▲); PHB(□); xylitol(○); acetate(◊).

As acetate levels were high and xylose utilization stopped before depletion, PHB production was also evaluated in another set-up using well-aerated and pH-controlled bioreactor for strain TMB 4443 (PHB+). Under these conditions, full xylose consumption was observed within 48 hours (Figure 3) and biomass accumulation was very high in comparison to the shake flask-experiments. Also, acetate, ethanol and xylitol production was significantly reduced. At the end of the exponential phase (48 hours), maximum biomass accumulation was reached and cellular PHB reached 0.49 ± 0.06% PHB/CDW, corresponding to a PHB yield of 1.99 ± 0.15 mg PHB g^−1^ xylose (Figure 3, Table 3).

## Discussion

In the present study, PHB production from xylose was demonstrated using recombinant *Saccharomyces cerevisiae.* PHB production occurred under exponential growth phase, concomitant with the accumulation of ethanol and other by-products. The maximal PHB yield from xylose (1.99 ± 0.15 mg PHB g^−1^ xylose, or 2.79 Cmmol PHB Cmol^−1^ xylose), that was achieved in well-aerated bioreactors, was comparable to reported non-xylose engineered strains harboring equivalent *C. necator* PHB pathways growing on glucose (Table 4), thereby demonstrating the potential of using a pentose carbon source, and more generally C5-rich lignocellulosic substrates for PHB production. One of the possible reasons might be that xylose conversion to pyruvate does not involve the oxidative pentose phosphate pathway that is the main source of NADPH inside the cell (Bruinenberg et al. 1983). As a consequence more carbon may be diverted towards the NADP^+^-catalyzed formation of acetate that is a direct precursor for PHB synthesis.

**Table 4:** **Comparison of selected PHB producing** ***S. cerevisiae*** **strains harbouring the** ***C. necator*** **PHB pathway, consuming different carbon sources**

**Strain**	**Genotype**	**Carbon source**	**Growth conditions**	**PHB yield/carbon source**		**PHB content (mg/g CDW)**	**Reference**
				**(mg/g)**	**(Cmmol/Cmol)**		
SCKK005	PHB pathway	Glucose	Shake flask	~1.5^a^	2.1^a^	4.02	(Kocharin et al. 2013)
SCK006	PHB pathway, *ADH2*, *ALD6*, *acs* ^L641P^, *ERG10*	Glucose	Shake flask	~5^a^	7^a^	15.89	(Kocharin et al. 2013)
SCK032	PHB pathway, *xpkA*, *ack*, *acs* ^L641P^	Glucose	Shake flask	~9^a^	12.5^a^	27.86	(Kocharin et al. 2013)
SCK034	PHB pathway, *gapN*, *ADH2*, *ALD6*, *acs* ^L641P^, *ERG10*	Glucose	Shake flask	~7^a^	9.8^a^	27.52	(Kocharin et al. 2013)
SCKK006	PHB pathway, *ADH2*, *ALD6*, *acs* ^L641P^, *ERG10*	Glucose	Aerobic, chemostat bioreactor, dilution rate 0.10 h^−1^	2.79	3.67	5.59	(Kocharin and Nielsen 2013)
Strain RKS	GAL1-10-induced PHB pathway	Galactose/Glucose	Aerobic, batch bioreactor	13.5^b^	18.8^b^	75	(Carlson and Srienc 2006)
TMB4443	PHB Pathway, *XYL1*, *XYL2*, *XKS*, PPP↑, *gre3*Δ	Xylose	Shake flask	1.17	1.63	10.4	This study
TMB4443	PHB Pathway, *XYL1*, *XYL2*, *XKS*, PPP↑, *gre3*Δ	Xylose	Aerobic, batch bioreactor	1.99	2.79	4.9	This study

^a)^Recalculated from graph presented in the respective references, based on total consumption of carbon sources.

^b)^Calculated based on total consumption of carbon sources.

**Figure 4:**
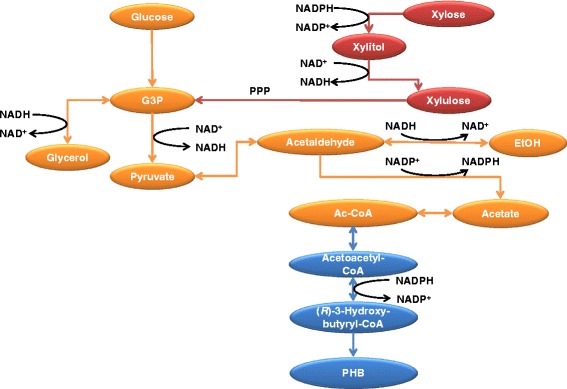
**A condensed view of the glycolytic pathway steps, as well as the XR and PHB pathway steps that are relevant for the PHB accumulation in the constructed strain.** Abbreviations: EtOH, ethanol; Ac-CoA, acetyl coenzyme A; G3P, glyceraldehyde 3-phosphate; PPP, pentose phosphate pathway The parts marked in red are the introduced xylose assimilation pathway and the overexpressed PPP. The parts marked in blue are the components of the PHB metabolic pathway.

The final titer reached a total of 45.0 ± 3.5 mg L^−1^ of PHB for TMB 4443 (PHB+) in shake flask conditions, and increased to 101.7 ± 7.1 mg L^−1^ of PHB in well-aerated bioreactor. By-product accumulation was much lower in the fully aerobic and pH-controlled bioreactor set-up than in the shake-flask experiment, indicating increased respiratory metabolism that directly translated into higher biomass. This may explain the positive impact on PHB production as more cells were available to synthesize PHB while less carbon was dissipated into reduced byproducts. This observation was made despite the fact that low levels of acetate, that is required for PHB production (Figure 4), were generated. In contrast, unusually high NADP^+^-catalyzed acetate levels were observed in the shake flask experiments. In that case, the limited respiration may trigger higher availability of acetaldehyde precursor; this, combined with the need for additional routes for NADPH generation for biomass synthesis and for xylose utilization via the NAD(P)H-dependent xylose reductase, could explain the dramatic increase in acetate production. It may also explain why xylose consumption stopped before completion as pH decreased over time (data not shown). The accumulation of acetate also indicates that, under these conditions, PHB generation may rather be controlled by reactions downstream of acetate (Figure 4). This includes the acetyl-CoA synthesis that has been shown to be limited by the availability of ATP and by the efficiency of the acetyl-CoA synthetase (Shiba et al. 2007), or the acetoacetyl-CoA reductase-catalyzed reduction that could be limited by NADPH shortage. Overall, the results indicate that a threshold in oxygenation may be necessary to provide sufficient resources for biomass synthesis while enabling sufficient formation and further conversion of the acetate precursor.

In this report PHB production from xylose was demonstrated in *S. cerevisiae.* Our results also point towards the importance of aeration on the synthesis of the PHB pathway precursor and cofactors. To further enhance the PHB accumulation process aspects such as redox balance and the acetyl-CoA node have to be considered.

## Abbreviations

Acetyl-CoA: Acetyl coenzyme A
NAD^+^/H: Nicotinamide adenine dinucleotide
NADP^+^/H: Nicotinamide adenine dinucleotide phosphate
PCR: Polymerase chain reaction
OE-PCR: Overlap extension PCR
XR: Xylose reductase

## References

[CR1] Bengtsson O, Hahn-Hägerdal B, Gorwa-Grauslund MF (2009) Xylose reductase from *Pichia stipitis* with altered coenzyme preference improves ethanolic xylose fermentation by recombinant *Saccharomyces cerevisiae*. Biotechnol Biofuels 2. doi:910.1186/1754-6834-2-9 10.1186/1754-6834-2-9 PMC2688486 19416504

[CR2] Breuer U, Terentiev Y, Kunze G, Babel W (2002). Yeasts as producers of polyhydroxyalkanoates: genetic engineering of *Saccharomyces cerevisiae*. Macromol Biosci.

[CR3] Bruinenberg PM, Vandijken JP, Scheffers WA (1983). A theoretical analysis of NADPH production and consumption in yeasts. J Gen Microbiol.

[CR4] Carlson R, Srienc F (2006). Effects of recombinant precursor pathway variations on poly [(R)-3-hydroxybutyrate] synthesis in *Saccharomyces cerevisiae*. J Biotechnol.

[CR5] Demeke MM, Dietz H, Li Y, Foulquie-Moreno MR, Mutturi S, Deprez S, Den Abt T, Bonini BM, Liden G, Dumortier F, Verplaetse A, Boles E, Thevelein JM (2013). Development of a D-xylose fermenting and inhibitor tolerant industrial *Saccharomyces cerevisiae* strain with high performance in lignocellulose hydrolysates using metabolic and evolutionary engineering. Biotechnol Biofuels.

[CR6] European Parliament CotEU (2009). Promotion of the use of energy from renewable sources.

[CR7] Foley JA, Ramankutty N, Brauman KA, Cassidy ES, Gerber JS, Johnston M, Mueller ND, O’Connell C, Ray DK, West PC, Balzer C, Bennett EM, Carpenter SR, Hill J, Monfreda C, Polasky S, Rockström J, Sheehan J, Siebert S, Tilman D, Zaks DP (2011). Solutions for a cultivated planet. Nature.

[CR8] Gietz RD, Akio S (1988). New yeast-*Escherichia coli* shuttle vectors constructed with in vitro mutagenized yeast genes lacking six-base pair restriction sites. Gene.

[CR9] Gietz R, Schiestl R (2007). Quick and easy yeast transformation using the LiAc/SS carrier DNA/PEG method. Nat Protoc.

[CR10] Gírio F, Fonseca C, Carvalheiro F, Duarte L, Marques S, Bogel-Lukasik R (2010). Hemicelluloses for fuel ethanol: a review. Bioresour Technol.

[CR11] Hahn-Hägerdal B, Karhumaa K, Fonseca C, Spencer-Martins I, Gorwa-Grauslund MF (2007). Towards industrial pentose-fermenting yeast strains. Appl Microbiol Biotechnol.

[CR12] Karhumaa K, Hahn-Hägerdal B, Gorwa-Grauslund MF (2005). Investigation of limiting metabolic steps in the utilization of xylose by recombinant *Saccharomyces cerevisiae* using metabolic engineering. Yeast.

[CR13] Kim S, Park Y-C, Jin Y-S, Seo J-H (2013) Strain engineering of *Saccharomyces cerevisiae* for enhanced xylose metabolism. Biotechnol Adv. doi:10.1016/j.biotechadv.2013.03.004 10.1016/j.biotechadv.2013.03.004 23524005

[CR14] Kocharin K, Nielsen J (2013). Specific growth rate and substrate dependent polyhydroxybutyrate production in *Saccharomyces cerevisiae*. AMB Express.

[CR15] Kocharin K, Chen Y, Siewers V, Nielsen J (2012). Engineering of acetyl-CoA metabolism for the improved production of polyhydroxybutyrate in *Saccharomyces cerevisiae*. AMB Express.

[CR16] Kocharin K, Siewers V, Nielsen J (2013) Improved polyhydroxybutyrate production by *Saccharomyces cerevisiae* through the use of the phosphoketolase pathway. Biotechnol Bioeng. doi:10.1002/bit.24888 10.1002/bit.24888 23456608

[CR17] Lambert JG, Hall CAS, Balogh S, Gupta A, Arnold M (2014). Energy, EROI and quality of life. Energy Policy.

[CR18] Law J, Slepecky R (1961). Assay of poly-beta-hydroxybutyric acid. J Bacteriol.

[CR19] Leaf T, Peterson M, Stoup S, Somers D, Srienc F (1996). *Saccharomyces cerevisiae* expressing bacterial polyhydroxybutyrate synthase produces poly-3-hydroxybutyrate. Microbiology.

[CR20] Lian J, Si T, Nair NU, Zhao H (2014). Design and construction of acetyl-CoA overproducing *Saccharomyces cerevisiae* strains. Metab Eng.

[CR21] Madison LL, Huisman GC (1999). Metabolic engineering of poly(3-hydroxyalkanoates): from DNA to plastic. Microbiol Mol Biol Rev: MMBR.

[CR22] Nijkamp JF, van den Broek M, Datema E, de Kok S, Bosman L, Luttik MA, Daran-Lapujade P, Vongsangnak W, Nielsen J, Heijne WH, Klaassen P, Paddon CJ, Platt D, Kötter P, van Ham RC, Reinders MJ, Pronk JT, de Ridder D, Daran J-MMC (2012). De novo sequencing, assembly and analysis of the genome of the laboratory strain *Saccharomyces cerevisiae* CEN.PK113-7D, a model for modern industrial biotechnology. Microb Cell Fact.

[CR23] Novy V, Krahulec S, Longus K, Klimacek M, Nidetzky B (2013). Co-fermentation of hexose and pentose sugars in a spent sulfite liquor matrix with genetically modified *Saccharomyces cerevisiae*. Bioresour Technol.

[CR24] Philip S, Keshavarz T, Roy I (2007) Polyhydroxyalkanoates: biodegradable polymers with a range of applications. J Chem Technol Biotechnol 82. doi:10.1002/jctb.1667

[CR25] Poehlein A, Kusian B, Friedrich B, Daniel R, Bowien BC (2011). Complete genome sequence of the type strain *Cupriavidus necator* N-1. J Bacteriol.

[CR26] Runquist D, Hahn-Hägerdal B, Bettiga M (2010). Increased ethanol productivity in xylose-utilizing *Saccharomyces cerevisiae* via a randomly mutagenized xylose reductase. Appl Environ Microbiol.

[CR27] Shiba Y, Paradise EM, Kirby J, Ro DK, Keasing JD (2007). Engineering of the pyruvate dehydrogenase bypass in Saccharomyces cerevisiae for high-level production of isoprenoids. Metab Eng.

[CR28] Sun J, Shao Z, Zhao H, Nair N, Wen F, Xu J-H, Zhao H (2012). Cloning and characterization of a panel of constitutive promoters for applications in pathway engineering in *Saccharomyces cerevisiae*. Biotechnol Bioeng.

[CR29] Suriyamongkol P, Weselake R, Narine S, Moloney M, Shah S (2007). Biotechnological approaches for the production of polyhydroxyalkanoates in microorganisms and plants - a review. Biotechnol Adv.

[CR30] Vandamme P, Coenye T (2004). Taxonomy of the genus *Cupriavidus*: a tale of lost and found. Int J Syst Evol Microbiol.

